# Role of Histone Deacetylase 6 and Histone Deacetylase 6 Inhibition in Colorectal Cancer

**DOI:** 10.3390/pharmaceutics16010054

**Published:** 2023-12-29

**Authors:** Ana Vuletić, Katarina Mirjačić Martinović, Jelena Spasić

**Affiliations:** 1 Department of Experimental Oncology, Institute of Oncology and Radiology of Serbia, Pasterova 14, 11000 Belgrade, Serbia; katarina.mirjacic@ncrc.ac.rs; 2 Clinic for Medical Oncology, Institute of Oncology and Radiology of Serbia, Pasterova 14, 11000 Belgrade, Serbia; jelena.spasic@ncrc.ac.rs

**Keywords:** HDAC6, histone deacetylase inhibitors, colorectal cancer

## Abstract

Histone deacetylase 6 (HDAC6), by deacetylation of multiple substrates and association with interacting proteins, regulates many physiological processes that are involved in cancer development and invasiveness such as cell proliferation, apoptosis, motility, epithelial to mesenchymal transition, and angiogenesis. Due to its ability to remove misfolded proteins, induce autophagy, and regulate unfolded protein response, HDAC6 plays a protective role in responses to stress and enables tumor cell survival. The scope of this review is to discuss the roles of HDCA6 and its implications for the therapy of colorectal cancer (CRC). As HDAC6 is overexpressed in CRC, correlates with poor disease prognosis, and is not essential for normal mammalian development, it represents a good therapeutic target. Selective inhibition of HDAC6 impairs growth and progression without inducing major adverse events in experimental animals. In CRC, HDAC6 inhibitors have shown the potential to reduce tumor progression and enhance the therapeutic effect of other drugs. As HDAC6 is involved in the regulation of immune responses, HDAC6 inhibitors have shown the potential to improve antitumor immunity by increasing the immunogenicity of tumor cells, augmenting immune cell activity, and alleviating immunosuppression in the tumor microenvironment. Therefore, HDAC6 inhibitors may represent promising candidates to improve the effect of and overcome resistance to immunotherapy.

## 1. Introduction

Colorectal cancer (CRC) is the third most common cancer and the second in terms of the cause of cancer-related deaths, which is mostly due to the establishment of diagnosis at advanced stages of the disease [1]. CRC is a heterogeneous disease characterized by variations in molecular profiles and clinical manifestations; therefore, the treatments for CRC are based on the histopathological type and clinical stage of the disease [2]. Currently, for clinical stage I and stage II CRC, surgical resection of the primary tumors shows high therapeutic success, with or without adjuvant radio-chemotherapy for high-risk patients in clinical stages II and III. Unfortunately, patients in stage III CRC usually suffer from the recurrence of the disease, which is often associated with micrometastasis. Patients with metastatic disease in clinical stage IV of CRC are treated with oxaliplatin/irinotecan, folinic acid, and 5-fluorouracil (5-FU)-based chemotherapeutic regimens [2]. However, as the treatment of these patients is often followed by drug resistance and subsequent disease progression, novel therapeutic options targeting oncogenic signaling pathways have been introduced in combination with chemotherapy. Testing for KRAS and NRAS exons 2, 3, and 4 as well as BRAF mutations is recommended for these patients, due to its relevance in selecting therapy. In this sense, these mutations are negative predictive factors for the use of therapeutic anti-epidermal growth factor receptor (EGFR) monoclonal antibodies. The *BRAF*V600E mutation is a strong negative prognostic factor in metastatic CRC [2,3]. Furthermore, anti-angiogenic drugs are approved for the treatment of the metastatic stage of CRC [4], whereas the application of immunotherapy is still limited to microsatellite instability-high (MSI-H) tumors [5]. Unfortunately, in patients with metastatic CRC, these therapeutic options have shown limited therapeutic effect and are often accompanied by disease progression. Therefore, the understanding of mechanisms involved in tumor development and progression of CRC is necessary for the identification of novel targets for new and combinational therapies. In recent years, histone deacetylases (HDACs) have been identified as relevant mechanisms that contribute to the pathogenesis and metastatic invasion of CRC [6,7].

HDACs were originally characterized as nuclear enzymes that catalyze the removal of acetyl groups from the ϵ-amino groups of lysine (Lys) residues in histone proteins and thereby regulate the higher-order chromatin structure and repress gene expression [8,9,10]. Together with their functional counterparts, the histone acetyltransferases (HATs) HDACs play a key role in epigenetic regulation of gene expression and are often dysregulated in multiple malignancies [6,7]. HDACs also deacetylate a variety of non-histone nuclear and cytoplasmic proteins [11]. Based on their structure and enzymatic activity, HDACs are divided into four major classes. Class I HDACs organize chromatin as the catalytic subunits within seven distinct multiprotein corepressor complexes and represent the established drug targets [12]. It was reported that HDAC1, HDAC2 [13], and HDAC3 [14] are up-regulated in colon cancer cells. Moreover, the expression of HDAC1 and 2 was associated with poor prognosis in colon cancer patients [15]. 

Class II HDACs are known to shuttle between the nucleus and the cytoplasm. HDAC6 is a class II subclass b HDAC, mainly localized in the cytoplasm of differentiated cells [16,17]. HDAC6 regulates signaling pathways that are involved in tumor cell growth, survival, and invasiveness and are often overexpressed in the majority of malignancies, including CRC. An increased expression of HDAC6 was reported in colon cancer tissue compared to the adjacent noncancerous tissue and is often associated with unfavorable disease prognosis [18,19]. 

**Figure 1:**
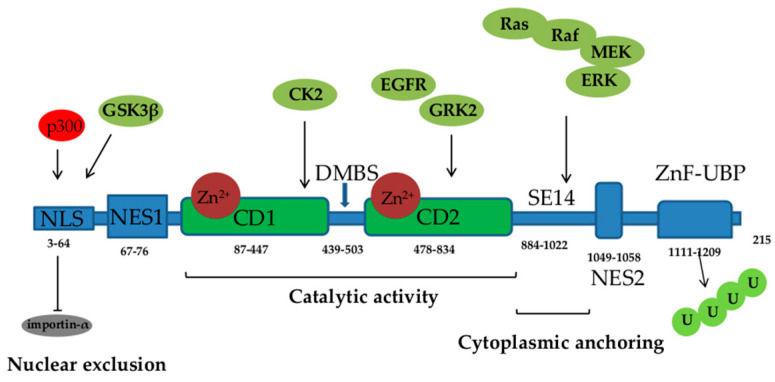
Functional domains of histone deacetylase (HDAC) 6: NLS—nuclear localization sequence, NES—nuclear export sequence; two catalytic domains (CD1 and CD2), DMBS—dynein motor-binding sequence, ZnF-UBP—zinc finger ubiquitin-binding domain, U—ubiquitin. Acetylation of NLS by p300 inhibits HDAC6 interaction with importin-α. Ras/Raf/mitogen-activated protein kinase (MEK)/extracellular signal-regulated kinase (ERK), epidermal growth factor receptor (EGFR), G protein-coupled receptor kinase (GRK) 2, casein kinase (CK) 2, and glycogen synthase kinase (GSK) 3β signaling phosphorylate and activate HDAC6.

HDAC6 has gained a lot of attention since its discovery in 1999 [20]. It consists of 1215 amino acids and possesses five functional domains (Figure 1). Starting from its N- to the C- terminus, HDAC6 comprises the following regions: a nuclear localization sequence (NLS) that is rich in arginine (Arg) and Lys, a nuclear export sequence (NES) that is rich in leucine (Leu), two catalytic deacetylase domains (CD1 and CD2), the cytoplasmic anchoring serin (Ser) glutamine (Glu)-containing tetrapeptide (SE14), and a ubiquitin-binding zinc finger motif domain (ZnF-UBP). NLS and NES together control the trafficking of HDAC6 between the nucleus and the cytoplasm, while SE14 is responsible for the intracellular retention of HDAC6. HDAC6 also contains a dynein motor-binding sequence (DMBS) between the CD1 and CD2 catalytic domains [17,20,21,22].

With the two catalytic domains, HDAC6 is a unique class IIb HDAC, responsible for the deacetylation of a number of non-histone substrates involved in the regulation of crucial physiological processes, including cell proliferation, survival, apoptosis, autophagy, motility, intracellular transport, and stress responses. HDAC6 regulates the deacetylation of multiple cytoplasmic substrates and affects their activity, cellular location, and protein–protein interactions. The deacetylase activity of both catalytic domains is Zn^2+^ dependent. The CD1 catalytic domain mostly deacetylates C-terminal acetyl-Lys residues and cannot independently exert catalytic activity but needs CD2 assistance. Aside from its deacetylase activity, HDAC6, through the catalytic CD1 domain, exhibits E3 ubiquitin ligase activity [21]. Proteins in the cytoplasm that are identified as substrates for deacetylation by HDAC6 include α-tubulin, cortactin, heat shock protein (Hsp) 90, heat shock transcription factor-1 (HSF-1), Ku70, p53, peroxiredoxins, signal transducer and activator of transcription (STAT) 3, forkhead box protein O1 (FOXO1), and β-Catenin [17,18,23,24,25] (Table 1).

HDAC6 is partially localized in the nucleus and interacts with histone H4 [18] and non-histone nuclear proteins, including HDAC11 [50,51], the transcriptional corepressor LCoR [51], and transcription factors such as nuclear factor-kappa B (NF-κB) and Runx [17,35]. Aside from its deacetylase activity, HDAC6 exhibits its biological function by nonenzymatic interactions with other proteins (Table 2). 

In this sense, via its ZnF-UBP domain, HDAC6 binds to ubiquitin as well as to sequence-specific proteins via its C-terminal glycine (Gly)–Gly motif that regulates cellular functions such as autophagic degradation, formation, and assembly of inflammasomes [59].

The catalytic activity of HDAC6 is affected by its subcellular localization and regulated by posttranslational mechanisms such as phosphorylation and acetylation that increase its activity. Phosphorylation of threonine (Thr), Ser, or Tyr residues of HDAC6 by various kinases (including extracellular signal-regulated kinase (ERK), glycogen synthase kinase (GSK) 3β, G protein-coupled receptor kinase (GRK) 2, protein kinase C (PKC)) and growth factor receptors such as epidermal growth factor receptor (EGFR) augments its enzymatic activity [17,60]. Furthermore, HDAC6 activity can be regulated by modifications of Lys residues, including SUMOylation, ubiquitination, and acetylation. Although the biological consequence of SUMOylation is not known, recent evidence shows that ubiquitination may alter the activity of the target protein without inducing its degradation [61], while Lys acetylation attenuates the enzymatic activity of HDAC6 [52,62]. The predominantly cytoplasmic localization of HDAC6 is influenced by NES and this process is dependent on the chromosome region maintenance protein 1 (CRM1), which is the main protein receptor that facilitates the export of molecules from the nucleus to the cytoplasm [63,64]. Furthermore, the acetylation of the NLS sequence of HDAC6 by histone acetyltransferase p300 contributes to the retention of HDAC6 in the cytoplasm as it blocks the interaction with the nuclear import protein importin-α [26]. 

**Figure 2:**
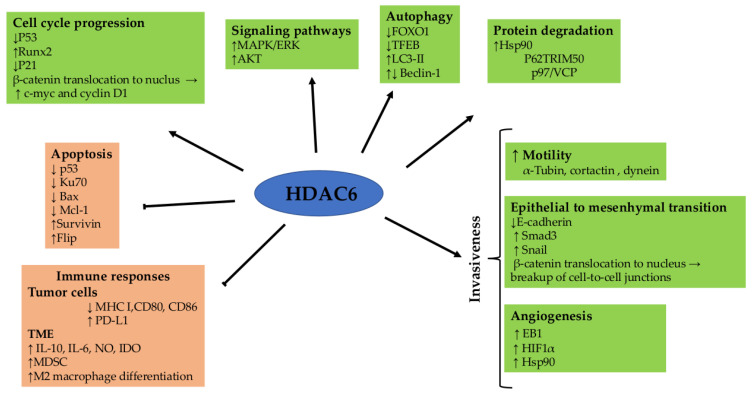
Roles of histone deacetylase (HDAC) 6 in tumorigenesis and tumor progression. HDAC6 induces cell cycle progression, activation of mitogen-activated protein kinase (MEK)/extracellular signal-regulated kinase (ERK), and protein kinase c AKT signaling pathways, autophagy, degradation of defective proteins, and tumor invasiveness by increasing cell motility, epithelial to mesenchymal transition, and angiogenesis, while inhibiting apoptosis and suppressing immune responses by decreasing immunogenicity of tumor cells and inducing immunosuppression in tumor microenvironment (TME). TFEB—transcription factor EB, LC—microtubule-associated protein 1 light chain 3, Hsp—heat shock protein 90, EB—end-binding protein 1, HIF—hypoxia-inducible factor 1α, MHC—major histocompatibility class I molecule, PD-L1—programmed death-ligand 1, IL—interleukin, NO—nitric oxide, IDO—indoleamine 2,3-dioxygenase, MDSC—myeloid-derived suppressive cells.

HDAC6 is involved in a plethora of physiological processes (Figure 2) that are often dysregulated in CRC [6,7].

## 2. Roles of HDAC6 in Physiological Processes

### 2.1. Cytoskeleton Organization

HDAC6 affects cytoskeletal structure and dynamics by modifying microtubule and actin organization and thereby is involved in the maintenance of cellular shape, cell division, cell migration, intracellular transport, and angiogenesis. The cytoskeletal component, α-tubulin, is acetylated at Lys40 by α-tubulin acetyltransferase (αTAT), which enables tubulin polymerization and formation of microtubules, while its deacetylation by HDAC6 promotes microtubule disassembly [27,28]. The overexpression of HDAC6 is associated with tubulin deacetylation, proclivity to chemotactic movement, and increased cell motility [29]. Furthermore, HDAC6 deacetylates another cytoskeletal protein cortactin that is present in areas of dynamic actin assembly such as at the leading edge of migrating cells. Deacetylated cortactin subsequently binds to F-actin through the small GTPase Rac1 and the actin nucleating complex Arp2/3, which enhances actin polymerization and branching, leading to increased cell motility. Cortactin is often overexpressed in tumors [65], including CRC, where it promotes malignant cell proliferation by activating the EGFR–MAPK pathway. Its expression was reported to correlate with the metastatic potential of a tumor [30,66]. The acetylation of cortactin prevents its translocation to the cell periphery, blocks its association with F-actin, and impairs cell motility, while its deacetylation by HDAC6 increases tumor cell motility and hence their invasiveness [26,30].

### 2.2. Degradation of Damaged Proteins

HDAC6 mediates the clearance of misfolded and damaged proteins that are often accumulated in malignant cells. Although the majority of defective proteins are labeled with ubiquitin chains and degraded by the ubiquitin–proteasome (UP) pathway, if the proteasome system is overwhelmed or if protein aggregates are unsuitable for UP degradation, they are degraded in autophagosomes. Cancer cells are characterized by extensive autophagic activity that modulates the recycling of cellular proteins, regulates cellular homeostasis and energy metabolism, enables uncontrollable proliferation, protects cells from stress imposed by accumulated damaged proteins, and promotes the survival of malignant cells [67]. 

HDAC6, through its UBP domain, simultaneously interacts with polyubiquitinated proteins while its DMBS interacts with the p150glued component of the dynein motor complex [27,54] (Table 2), and in this manner, it facilitates the transport of aggregated or misfolded proteins toward the microtubule-organizing center (MTOC) [68]. Furthermore, HDAC6 mediates the formation of aggresomes around the misfolded protein by deacetylating cortactin, which subsequently interacts with F-actin, triggering actin polymerization and fusion of aggresomes with autophagosomes and lysosomes to lyse proteins [69]. The ability of HDAC6 to mediate the formation and removal of aggresomes is regulated by casein kinase (CK) 2, which phosphorylates HDAC6 and increases its deacetylase activity [70].

HDAC6 coordinates the UP system and autophagy maintaining them in a complementary relationship by interacting with the scaffolding protein sequestosome 1 (SQSTM1)/P62. P62 functions as a ubiquitin-recognizing receptor that binds ubiquitinated proteins and encapsulates them into aggresomes, where they fuse with lysosomes and become degraded [71]. Furthermore, the E3-ubiquitin ligase TRIM50 promotes the recruitment and aggregation of polyubiquitinated (poly-U) proteins into aggresomes by facilitating the interactions of HDAC6 and p62 [53]. In this way, HDAC6 improves the efficiency and selectivity of autophagic degradation [72,73]. While TRIM50 promotes the clearance of ubiquitinated proteins in aggresomes, an HDAC6-interacting chaperone, valosin-containing protein p97/(VCP) ATPase, induces the dissociation of HDAC6 and poly-U proteins and protein delivery to proteasomes. In this sense, the excess of p97/VCP favors protein degradation in proteasomes [55]. It has been established that p62 and ubiquitin are highly expressed in colon carcinoma and that high ubiquitin expression has an impact on the number of lymph node metastases in patients with CRC [74]. Although, in some tumors, the expression of cytoplasmic p62 was negatively associated with patients’ survival, in CRC, a favorable prognostic significance of cytoplasmic p62 was found in the mutated K-RAS but not in the wild-type (wt) K-RAS subgroup of patients [75]. 

### 2.3. Autophagy

Aside from the formation of aggresomes and the transportation and degradation of autophagosomes, HDAC6 regulates autophagy by deacetylating autophagy-related transcription factors and proteins [76]. In this sense, HDAC6 deacetylates transcription factor EB (TFEB) [31] and forkhead box O1 (FOXO1) [32] to decrease their activity and to inhibit autophagy. TFEB and FOXO transcription factors are inactivated by phosphorylation [77]. HDAC6 inhibition promotes the acetylation of TFEB, which then enhances the expression of the autophagy-related protein Beclin-1 [78]. However, there are opposing data on the effect of HDAC6 on Beclin-1 expression obtained on liver cancer cells, showing that HDAC6 overexpression activated c-Jun NH2-terminal kinase (JNK) and increased the phosphorylation of c-Jun, which induced Beclin-1-dependent autophagy [79,80]. The acetylated FOXO1 transcription factor is also required for T-cell differentiation into regulatory T (Treg) cells expressing Foxp3 transcription factors [81]. It was reported that the pharmacological inhibition of HDAC6 enhanced the transcriptional activity of acetylated FOXO1 and facilitated the autophagy process [32,80].

Furthermore, HDAC6 regulates the activity of an autophagy-related protein, the microtubule-associated protein 1 light chain 3 (LC3). LC3-I forms a conjugate with phosphatidylethanolamine (PE) and becomes LC3-II, which is then transported by HDAC6 to the MTOC to promote autophagosome formation [80,82]. It was reported that the deacetylation of LC3-II by HDAC6 promotes its translocation to the cytoplasm and autophagy, thereby inducing the survival of nutrient-deprived tumor cells [80,83]. This suggests that HDAC6 functions both as a scaffold protein and as a deacetylase in the regulation of LC3 that promotes autophagy [80]. Cytoskeletal modifications by HDAC6 are relevant for the progression of autophagy. While the actin remodeling induced by cortactin deacetylation by HDAC6 is essential for the fusion of autophagosomes and lysosomes [69], the deacetylation of α-tubulin and microtubule disassembly inhibits their fusion with lysosomes [84].

It was reported that the expression of nuclear Beclin-1 and LC3 in patients with CRC harboring K-RAS mutations is associated with shorter overall survival [75]. Moreover, according to one study, the activation of the KRAS/BRAF/phosphoinositide 3-kinase (PI3K) oncogenic pathway by *KRAS* and *BRAF*V600E mutations induces the expression of the key autophagic markers lLC3 and Beclin-1 in CRC cells, thus promoting autophagy [85].

### 2.4. Regulation of Molecular Chaperones and Other Stress-Related Proteins

HDAC6 affects protein degradation in proteasomes by the deacetylation of the Hsp90 molecular chaperone that has a primary physiological ability to stabilize protein tertiary structures, regulate transportation, and prevent protein degradation in proteasomes, thus enabling the biological functions of its client protein [56]. Multiple client proteins are regulated by Hsp90, such as steroid hormone receptors, growth factor receptors (EGFR, vascular endothelial growth factor (VEGFR)), molecules engaged in the regulation of the cell cycle and apoptotic pathways, transcription factors such as hypoxia-inducible factor 1-α (HIF-1α), signaling molecule RAF Ser/Thr protein kinase, etc. The acetylation of Hsp90 impairs its chaperone activity and therefore induces degradation of the client protein. Simultaneously, the stability of HDAC6 is modulated by Hsp90 [33], while the degradation of HDAC6 is regulated by cullin 3^SPOP^ ubiquitin E3 ligase that has been shown to promote HDAC6 polyubiquitination and degradation in proteasomes in multiple cancer cell lines, including CRC [86].

HDAC6 also regulates the function of Hsp90 through the ZnF-UBP domain. HDAC6 senses ubiquitinated protein aggregates but also indirectly induces the expression of molecular chaperones, as it can activate p97/VCP that through its enzymatic activity induces the release of HSF1 from the Hsp90/HSF1 complex [54,55]. The released HSF1 induces the expression of genes for molecular chaperones Hsp70 and Hsp90 [57], which is followed by the release of HDAC6 from the complex and its binding to ubiquitinated proteins [56,67].

In CRC, the expression of Hsp90 in tumor tissue was inversely associated with survival outcomes and could represent a potential unfavorable prognostic factor for CRC patients [87]. As HDAC6 protects tumor cells from cytotoxic effects caused by defective and misfolded proteins, the inhibition of HDAC6 and Hsp90 may have therapeutic potential in cancer. In this sense, inhibition of Hsp90 has shown an antitumor effect in animal models of CRC and in CRC cell lines as it caused the depletion of B-RAF and K-RAS, which are major oncogenic drivers in CRC associated with poor disease prognosis [88].

HDAC6 plays an active role in the response to environmental stress imposed by newly synthesized secretory proteins in the endoplasmic reticulum (ER) in order to eliminate misfolded proteins before protein aggregation becomes lethal for the cell [89]. In this sense, HDAC6 targets the 78 kDa glucose-regulated protein (GRP78) and Hsp70, the molecular chaperones that are involved in the unfolded protein response (UPR) that implies the regulation of proper folding, conformational maturation, assembly of proteins in ER, and control of the overall quality of proteins. Aside from its role in the UPR, GRP78 displays antiapoptotic properties, promotes tumor proliferation, survival, and metastasis, and confers resistance to chemotherapy. The level of GRP78 was inversely associated with the sensitivity of CRC cells to alkylating agents, including cisplatin and 5-FU [90]. In solid tumors, hypoxic conditions, acidosis, and glucose deficiency induce GRP78 expression [91]. It has been shown that colon cancer cells secrete GRP78 via exosomes and that this process is dependent on the activity of HDAC6 [34], which is often overexpressed in CRC [19]. HDAC6 inhibition increases GRP78 acetylation. Subsequently, the acetylated GRP78 dissociates from HDAC6 and then binds to VPS34, a class III PI3K, thus preventing the sorting of GRP78 into multivesicular bodies and GRP78 release that induces its aggregation in the ER that further inhibits tumor growth [34]. Moreover, by inhibiting the release of exosomes containing GRP78 from cancer cells, HDAC6 inhibition also inhibits angiogenesis as GRP78 is involved in blood vessel formation in growing tumors through the activation of HIF-1α and VEGF/VEGFR, as well as the PI3K/AKT, ERK, and FAK signaling pathways [92].

HDAC6 has an important role in redox regulation in response to cellular stress. HDAC6 deacetylates the redox-regulatory antioxidant enzymes peroxiredoxin (Prx) I and Prx II [42]. Prxs are often present at high levels in cancer and neurodegenerative disorders and play a protective role against oxidative damage. The acetylation of Prx increases its reductase activity, thus, HDAC6 and Prx may be considered as therapeutic targets for modulating intracellular redox status in cancer [22].

### 2.5. Apoptosis

HDAC6 affects cell cycle progression and apoptosis by modulating the activity of multiple proteins, including p53. Upon DNA damage, p53 is activated by the kinases ATM, ATR, Chk1, and Chk2. This leads to the disruption of the interaction between p53 and mouse double minute 2 homolog (MDM2), which is an E3 ubiquitin-protein ligase [93,94]. p53 can be acetylated by the acetyltransferases CBP and p300, which prevent its ubiquitination and enhance its stability and transcriptional activity toward the expression of proapoptotic proteins Bax and Puma [36,37,95]. Moreover, acetylated p53 releases the apoptotic molecule Bax from the nuclear p53/Bax complex, which is then translocated to the mitochondria to induce cytochrome C release and apoptosis [94,95]. HDAC6 has been found to deacetylate p53 and repress its function as a tumor suppressor [36,37,38]. It was reported that HDAC6 inhibition increases the acetylation of p53 in tumor cells, which leads to upregulated expression of genes related to cell cycle control and apoptosis, including p21 cyclin-dependent kinase (CDK) inhibitor, which can be induced with HDAC inhibition in p53-dependent and -independent ways [37,96]. Moreover, acetylated cytoplasmic p53 inhibits autophagy by inducing Beclin-1 degradation via the ubiquitin-specific peptidase USP10 and by inhibiting the mTOR (mechanistic target of rapamycin complex) pathway [38]. HDAC6 can also interact with p53 and attenuate its transcriptional activity through the promotion of its degradation [37].

p53 is mutated in 43% of CRC cases [97], indicating that the induction of the degradation of mutant p53 may represent a potential therapeutic approach. In this sense, several Hsp90 and HDAC inhibitors have been shown to destabilize p53 mutant proteins. Hsp90 inactivates the p53 E3 ubiquitin ligases MDM2 and CHIP, thereby increasing mutant p53 levels, while Hsp90 chaperone activity is enhanced by HDAC6-mediated deacetylation [98,99]. Thus, targeting of Hsp90 or HDAC6 induces the degradation of mutant p53. Whereas several Hsp90 inhibitors have so far only been investigated in clinical trials, HDAC inhibitors have already been approved by the Food and Drug Administration (FDA) for use in cancer therapy [37,97,100].

Due to the increased demand for the degradation of proteins in cancer, proteasomes play an important role in the maintenance of homeostasis. It has been reported that during the progression of CRC, the level of ubiquitin-conjugating enzymes (E2) that are involved in various tumor-promoting processes, specifically the newly identified UBE2Q1, is elevated. UBE2Q1 suppresses the transcriptional activities of p53 by inducing its ubiquitination and degradation and may thereby contribute to the survival of tumor cells [101].

Due to frequent p53 mutations in CRC, the apoptotic effect of HDAC inhibition is not always p53 dependent [102]. Recent studies indicate that the deacetylation of the DNA repair protein Ku70 by HDAC6 induces its binding to proapoptotic proteins Bax and Mcl-1 in the cytoplasm, increasing their stability and protecting cells from apoptosis [103]. However, in vitro treatments of CRC cell lines with HDAC6 inhibitors resulted in increased acetylation of Ku70 and induction of apoptosis by releasing Bax, which was subsequently translocated to mitochondria and induced cytochrome release [25,104]. Interestingly, the dissociation of the complexes formed between Ku70 and antiapoptotic FLIP protein following the acetylation of Ku70 was found to trigger FLIP polyubiquitination and degradation in proteasomes in CRC cells [39]. Furthermore, HDAC6 regulates apoptosis by the deacetylation of the antiapoptotic protein survivin, promoting its exit from the nucleus, which inhibits apoptosis of colon cancer cells [40,41].

Runt-related transcription factor-2 (Runx2), although initially defined as a transcription factor responsible for osteogenic differentiation in mammals, is closely related to proliferation, invasion, and bone metastasis of multiple cancer types. Interaction between Runx2 and HDAC6 leads to the recruitment of HDAC6 from the cytoplasm to chromatin and repression of the p21 gene promoter that induces proliferation [58]. In CRC cells with high Wnt signaling activity, Runx2 was designated as a critical transcription factor to trigger the expression of genes that regulate the epithelial-to-mesenchymal transition in vitro through the orchestration of chromatin organization [105]. Moreover, clinical data showed that Runx2 is closely related to an advanced stage of disease and liver metastasis in CRC patients and is associated with shorter survival [106].

### 2.6. Regulation of Signal Transduction Molecules

HDAC6 is also involved in the regulation of the RAS/RAF/MEK/ERK and PI3K/AKT/mTOR signaling pathways. It was first discovered that ERK1 phosphorylates and activates HDAC6 [60]. Further investigations revealed that HDAC6 deacetylates ERK1/2 and augments its activity while acetylation by CREB-binding protein and p300 decreases its activity toward the transcription factor ELK1. Accordingly, HDAC6 inhibition has been shown to suppress tumor proliferation and induce apoptosis via the deactivation of AKT and ERK signaling [48]. Moreover, HDAC6 affects AKT signaling indirectly by deacetylating Hsp90, which subsequently binds to AKT, protecting it from phosphatases and preserving its activity. By stimulating the AKT signaling pathway, HDAC6 contributes to cancer cell migration and angiogenesis [107]. HDAC6 inhibition has been shown to decrease AKT binding to PIP3 and activity [49]. This is relevant for CRC as this tumor may show increased activation of this signaling pathway as well as increased expression of HDAC6 [108].

HDAC6 regulates EGFR endocytosis and degradation by controlling the acetylation status of α-tubulin and subsequently receptor trafficking along microtubules. A negative feedback loop consisting of EGFR-mediated phosphorylation of HDAC6 on Tyr570 reduces the deacetylase activity and increases the acetylation of α-tubulin [109].

### 2.7. Regulation of NLRP3 Inflammasome

HDAC6 is involved in the assembly, priming, and activation of inflammasomes, the cytoplasmic protein complexes that are a crucial part of the innate immune system. The NLRP3 inflammasome contains an NLRP3 pattern recognition receptor (PRR), an apoptosis-associated spike-like protein containing a caspase recruitment domain for caspase-1 (ASC), and caspase-1 itself. In response to the activation of PRR by the products of damaged or dying cancer cells and activation of the NF-κB transcription factor, inflammasomes induce expression of NLRP3, pro-IL-1β, and pro-IL-18 [110]. This is followed by caspase-1-induced maturation of the proactive inflammatory cytokines to IL-1β and IL-18 and subsequent cleaving of gasdermin D to induce pyroptosis [80]. HDAC6 facilitates the priming of the NLRP3 inflammasome most prominently by deacetylating and activating the p65 subunit of NF-κB that subsequently induces the transcription of genes for NLRP3, pro-IL-1β, and pro-IL-18 [111]. Moreover, HDAC6 induces the activation of inflammasomes by activating PrxII, which increases the level of reactive oxygen species, which are important activators of inflammasomes [112]. It has been shown that HDAC6 inhibition upregulates p65 expression in the cytoplasm and reduces p65 expression in the nuclei of macrophages to attenuate the transcription of NLRP3 and reduce pyroptosis [113]. However, HDAC6 negatively regulates inflammasome activation through its interaction with ubiquitinated NLRP3 [80].

NLRP3 can impact CRC development due to its broad activity in shaping immune responses, apoptosis, and the gut microbiome. In this sense, the role of inflammasomes in colitis and colitis-associated CRC has been shown in animal models [114,115]. Moreover, the activation of the NLRP3 inflammasome in macrophages has been shown to promote the invasion of CRC cells by regulating the epithelial–mesenchymal transition via secretion of IL-1β from activated macrophages [115,116]. This finding, together with a clinical finding that suggested the positive correlation of NLRP3 expression with advanced disease and poor prognosis in patients with CRC, indicates the potential relevance of NLRP3 inflammasome as a therapeutic target [117].

### 2.8. Role of HDAC6 in Tumor Invasiveness

HDAC6 is involved in multiple phases of the epithelial–mesenchymal transition (EMT) by which tumor cells lose epithelial characteristics, cell-to-cell junctions, reorganize their cytoskeleton, increase cell motility, and acquire properties that are typical of mesenchymal cells. HDAC6 plays an important role in the EMT by deacetylating α-tubulin and augmenting cell motility. Moreover, a high level of acetylated α-tubulin was correlated with epithelial morphology, while the deacetylated form corresponded to EMT transition [118].

The most potent inducer of EMT, transforming growth factor (TGF)-β, has the ability to activate HDAC6. TGF-β and HDAC6 pathways intercept and induce phosphorylation and activation of the Smad3 molecule that inhibits the transcription of E-cadherin, leading to a mesenchymal-like phenotype of malignant cells [43]. It has been suggested that HDAC6 may induce the activity of the signal transducer Smad3 molecule directly by deacetylation [44], or indirectly by deacetylating α-tubulin and promoting Smad3 release [119]. Moreover, selective HDAC6 inhibition was reported to downregulate the expression of TGF-βRⅠ and the phosphorylation of Smad3 and EMT-inducing transcription factor Snail that led to the preserved expression of E-cadherin in cultured cancer cells [118,120].

IL-6 has been reported to induce the expression of HDAC6, concomitantly with increased proliferation, migration, and EMT of tumor cells. Moreover, the IL-6-induced HDAC6 not only upregulated the IL-6 downstream JAK2/STAT3 pathway but also co-activated TGF-β/Smad3 signaling [121]. The increased level of circulating IL-6 has been related to metastatic disease and poor prognostic outcome in cancer patients with diverse histological tumor types, as reported in numerous studies [122,123,124].

Through deacetylation of cortactin and tubulin, HDAC6 is involved in the formation of invadopodia, the actin-rich proteolytic structures specialized in the degradation of the extracellular matrix that mediates the invasion of malignant cells to distant tissues and organs [125]. Invadopodia have been observed during metastatic invasion of CRC [126]. The hypoxic conditions that prevail in solid tumors enhance HDAC6 deacetylase activity by EGFR, resulting in enhanced Smad phosphorylation and nuclear accumulation that influence invadopodia formation [127,128]. Therefore, considering the role of HDAC6 in hypoxia-induced metastatic invasion to regional lymphatics, the therapeutic targeting of HDAC6 may have important therapeutic implications for the treatment of metastatic disease [128,129].

Due to its role in the regulation of cytoskeletal dynamics, HDAC6 contributes to angiogenesis by regulating the polarization and migration of vascular endothelial cells in a microtubule end-binding protein (EB) 1-dependent manner and generating capillary-like structures [130]. In support of this, the upregulation of HDAC6 mRNA levels and protein levels has been shown in endothelial cells under hypoxic conditions [131]. Furthermore, HDAC6 was found to associate with HIF-1α [132] and with its transcriptional target, the VEGF receptor, thereby increasing their stability and activity in cancer cells [133]. Moreover, it was reported that HDAC6 affects the Hsp90-mediated regulation of VEGFR in tumor cells [134].

HDAC6 modifies β-catenin, which plays an essential role in cell-to-cell adherens junctions as it links E-cadherin to actin filaments. β-catenin is a key player in the Wnt cascade signaling pathway that induces EMT, cancer cell motility, and cancer stem cell maintenance [135]. The activation of HDAC6 upon simulation of EGFR leads to the deacetylation of β-catenin as well as the breakup of cell-to-cell junctions, which increases the level of nuclear β-catenin, either by direct release of the junctional β-catenin from the cell membrane or by activating E-cadherin endocytosis. Nuclear localization of β-catenin increases the proliferative potential of tumor cells by activation of target genes for c-myc and cyclin D1 [45]. It was reported that HDAC6 negatively regulates EGFR endocytosis and degradation in lysosomes by controlling the acetylation status of α-tubulin and hence the receptor trafficking along microtubules. However, the phosphorylation of HDAC6 by activated EGFR was found to reduce deacetylase activity and create a negative feedback loop, leading to increased degradation of activated EGFR [109]. Wnt/β-catenin signaling is involved in the tumorigenesis of CRC. The presence of mutations in APC (adenomatous polyposis coli) induces the nuclear localization of β-catenin and expression of Wnt target genes that promote tumor progression in CRC [45,136].

### 2.9. Involvement of HDAC6 in Immune Responses

HDAC6 has been shown to intervene in many aspects of the innate and adaptive immune responses. It affects antigen (Ag) uptake and presentation by antigen-presenting cells (APCs), dendritic cells (DCs), or macrophages and the cytotoxic function of natural killer (NK) cells, thus influencing the innate immune cells. Furthermore, HDAC6 partially affects T-cell activation and antitumor cytotoxicity in adaptive immune responses [137].

HDAC6 displays many of its immune-related effects by affecting the STAT3 signaling pathway, which is involved in the development of malignancies and in the induction and maintenance of immune tolerance and inhibition of immune responses [46,137]. STAT3 signaling can be activated with cytokines IL-6, IL-10, IL-21, and TNF that in DCs downregulate the expression of MHC class II and costimulatory molecules, thereby inducing the tolerogenic immune response [138]. Furthermore, STAT3 plays a key role in suppressing signal transduction mediated by Toll-like receptors (TLRs) in mature phagocytic cells. Accordingly, it has been shown that Stat3-deficient macrophages and DCs produce increased levels of proinflammatory cytokines (TNF, IL-1β, IL-6, IL-12) which activate the immune response upon TLR4 activation while reducing the amount of anti-inflammatory IL-10 and losing responsiveness to this cytokine that inhibits TLR4-dependent pro-inflammatory cytokine production [139]. The relevance of HDAC6–STAT3 signaling in immunomodulatory pathways in CRC has been confirmed by the pharmacological inhibition of HDAC6 that led to reduced functionality of STAT3 signaling, impacting the expression of genes involved in the inflammatory and immune responses [140].

HDAC6 is also involved in the regulation of macrophages [141]. In this sense, after stimulation of macrophages with lipopolysaccharide (LPS), HDAC6 was shown to translocate to the cell periphery where it induced cortactin deacetylation and the formation of invadopodia protrusions that increase cell mobility and enable infiltration into tissues [142]. Under physiological conditions, macrophages activated by LPS, IFN-γ, or TNF polarize into the M1 type that secretes pro-inflammatory cytokines (TNF, IL-1β, IL-6, and IL-12) and activate the antitumor immune response. Conversely, in tumors, after exposure to immunosuppressive cytokines IL-10 and TGF-β in the tumor microenvironment (TME), macrophages often differentiate into the M2 type, which is known to produce high concentrations of the immunosuppressive cytokine IL-10 [137]. However, HDAC6 inhibition in macrophages and DCs results in diminished production of IL-10 and enables them to effectively activate Ag-specific naive T cells. HDAC6 forms a molecular complex with STAT3 that has been detected in cytoplasmic and nuclear compartments of APCs [46].

It has been reported that colon cancer specimens with high HDAC6 expression show increased infiltration of immunosuppressive M2 macrophages that can be attributed to HDAC6 activity [47]. In this setting, HDAC6 deacetylates TGF-β-activated kinase 1 (TAK1), which subsequently activates p38 MAPK, leading to phosphorylation and activation of A disintegrin and metalloproteinase-17 (ADAM17). ADAM17, through its proteolytic activity, is responsible for the shedding of IL-6 receptor [47] from the cell membrane, resulting in the release of soluble IL-6 receptor (sIL-6R). Aside from the classical IL-6 signaling that involves IL-6 ligation to membrane-bound IL-6R and gp130 transmembrane receptor dimerization, “IL-6 trans-signaling” is mediated by sIL-6R, which forms a complex with IL-6 and directly engages gp130 [121]. Since ADAM17 is more abundant in CRC cells compared to normal tissue, it contributes to increased levels of soluble IL-6R that promote M2 macrophage polarization [47,143].

As HDAC6 is involved in the intracellular trafficking of granules, HDAC6-deficient CD8+ cytotoxic T lymphocytes (CTLs) were reported to display defective in vitro cytolytic activity due to altered dynamics, inhibited transport of lytic granules to the immune synapse, and deficient exocytosis, while target cell recognition, T cell receptor (TCR) activation, and IFN-γ production were not inhibited [144].

HDAC6 affects the development and activity of regulatory T cells (Treg) that have the physiological function of suppressing excessive immune responses to maintain immune homeostasis. In tumor immunity, Tregs are involved in tumor development and progression as they impair T cell function through the secretion of immune suppressive cytokines (IL-10, TGF-β, IL-35), consumption of IL-2 that leads to its depletion in the TME, and expression of inhibitory checkpoint receptor cytotoxic T lymphocyte-associated protein (CTLA-4). HDAC6 inhibits Treg differentiation as it deacetylates the Foxp3 transcription factor and inhibits the transcription of Foxp3-induced genes. It has been reported in several murine models that treatment with HDAC6-specific inhibitors increased the activity and induced the differentiation of Tregs [145,146]. Inhibition of HDAC6 by tubastatin A increased the acetylation of Hsp90 in Tregs, inducing the release of HSF-1 and upregulation of Treg-related genes [146]. Given that acetylation reduces the proteasomal degradation of FoxP3, HDAC6 inhibition would be expected to increase FoxP3 expression and increase the Treg number or function [147,148]. Regardless of these findings, there are conflicting reports on the role of HDAC6 in Treg cells. In this sense, one study reported that pharmacological inhibition of HDAC6 inhibited Treg cell differentiation and suppressive function in TGF-β-induced murine Treg cell differentiation by inhibiting their proliferation [149]. It has not yet been elucidated why different studies reported different effects of HDAC6 inhibition on Tregs. Increased accumulation of Treg cells is generally associated with CRC progression and metastasis, immunotherapy failure, and a poorer prognosis, although this correlation is not conclusive [150].

HDAC6 activity in tumor cells and immune cells in the TME has been shown to regulate the expression of tumor-associated antigens, MHC class I molecules, costimulatory molecules, and cytokines [151].

## 3. HDAC6 Inhibition in Colorectal Cancer

The oncogenic potential of HDAC6 has been well established in CRC as its inactivation by genetic manipulation reduced oncogenic transformation and tumor growth in in vitro as well as in vivo models [19,152]. More importantly, data obtained on HDAC6-null mice show that HDAC6 is not an essential gene for the development of an adult organism and that the physiological functions of normal cells are not affected by the deletion of the *HDAC6* gene [28,153]. Furthermore, it has been shown that, unlike the other HDACs, selective inhibition of HDAC6 impaired tumor growth and progression without inducing major adverse events in experimental animals [28,42,131,154]. Altogether, these characteristics make HDAC6 a highly desirable target for cancer treatment [155,156]. In experimental models of CRC, the *HDAC6* gene knockdown and pharmacological HDAC6 inhibition reduced cell viability and migration of tumor cells by inhibiting the MAPK/ERK pathway [19,152]. It has been reported that patients with CRC show significantly lower expression of SET7 Lys methyltransferase in cancer tissue than in adjacent tissue. SET7 catalyzes the methylation of the histone H3K4, which affects chromatin remodeling and regulates genes that are involved in cell cycle regulation, differentiation, and the DNA damage response and thereby plays a significant role in tumorigenesis. Moreover, SET7 functions as a tumor suppressor by inhibiting the deacetylating activity of HDAC6, partially through the ERK signaling pathway in colon cancer cells [152]. Downregulation of SET7 expression was closely correlated with poor prognosis in CRC [157], which is relevant for the oncogenic potential of HDAC6 in CRC. Furthermore, high expression of HDAC6 in CRC tissue was reported to be associated with reduced levels of acetylation at the 12th Lys residue of the histone H4 protein (H4K12ac). This histone residue was also reported to be highly sensitive to HDAC6 inhibition in several cancer types that subsequently induced chromatin relaxation [18].

HDAC inhibitors (HDACis) can be classified according to their chemical structure and their ability to inhibit the specific HDAC isoform or distinct HDAC classes. In this sense, HDAC6 activity can be inhibited by unselective or pan-HDACis that inhibit the majority of HDAC classes, and selective inhibitors that specifically target HDAC6 (HDAC6is).

Pan-HDACis have been extensively investigated in preclinical and clinical studies and have shown therapeutic benefits in hematological malignancies (multiple myeloma (MM), cutaneous T-cell lymphoma, peripheral T-cell lymphoma) that led to their approval for therapeutic applications. In this sense, suberoylanilide hydroxamic acid (SAHA, vorinostat) was the first approved pan-HDACi by the FDA, initially for the treatment of relapsed and refractory cutaneous T-cell lymphoma [158,159,160]. Following the successful clinical results with vorinostat, the pan-HDACis romidepsin and belinostat have been approved for the treatment of T-cell lymphoma and adult leukemia [161,162]. Another pan-HDACi, panobinostat (LBH589, Farydak), was approved and used in combination with the proteasome inhibitor bortezomib and the corticosteroid dexamethasone for the treatment of patients with recurrent MM and showed some therapeutic benefit [163].

Regarding the antitumor effect of pan-HDACis in CRC, in vitro studies have shown that treatment with vorinostat induced cell death of CRC cell lines (Table 3) regardless of the p53 mutational status and led to the downregulation of mutated p53 and upregulation of the wild-type (wt) p53. Moreover, a synergistic antiproliferative effect was shown when vorinostat was used in combination with the thymidylate synthase inhibitors 5-FU or raltitrexed, both commonly used in the treatment of CRC [164]. One study reported that vorinostat showed preferential cytotoxicity in cancer cells with mutated p53 by destabilizing mutant p53 through the inhibition of the HDAC6–Hsp90 chaperone axis [99]. Also, vorinostat showed apoptotic and antiproliferative effects on CRC cell lines in vitro in combination with another pan-HDAC inhibitor, trichostatin A. This treatment induced the attenuation of Wnt signaling due to proteasome-dependent degradation of the Wnt transcription factor TCF7L that was HDAC6 dependent [165].

Unlike clinical studies in hematological malignancies, in solid tumors, the use of a single pan-HDACi has led to limited therapeutic success due to considerable adverse effects. This was most likely the consequence of pharmacological targeting of more than one of the eighteen HDAC isoforms that affect multiple survival-related cellular pathways [179]. In this sense, a clinical trial showed that the pan-HDAC inhibitor romidepsin was ineffective in previously treated CRC patients with advanced disease [180]. Further clinical investigations evaluated the effect of HDAC inhibitors in combination with chemotherapy. Vorinostat was the first pan-HDACi to be used in a clinical trial in combination with conventional chemotherapy with 5-FU in patients with metastatic CRC who previously failed to respond to 5-FU-based chemotherapy [181] and in combination with leucovorin calcium in patients with recurrent CRC [182,183]. NCT numbers and references for the previously mentioned clinical trials are given in Table 4. Furthermore, a preclinical study showed improved antitumor activity of vorinostat in combination with the DNA methyl transferase inhibitor decitabine in a mouse model of CRC [166].

Aside from pan-HDAC inhibitors, selective HDAC inhibitors targeting other HDAC isoforms were investigated in clinical trials in patients with CRC in advanced stages of the disease, but mostly in combination with other therapeutics. In this sense, entinostat, the HDACi targeting 1, 2, and 3 isoforms, was investigated in a phase II clinical trial in combination with the pyrimidine nucleoside analog 5-azacitidine and showed improvement in overall survival of some patients [187]. The therapeutic potential of entinostat was also evaluated in combination with regorafenib, an antiangiogenic oral tyrosine kinase inhibitor and the inhibitor of autophagy hydroxychloroquine in a phase I trial (NCT03215264) to determine their effectiveness and tolerability in refractory advanced CRC [188]. Furthermore, another HDAC1,2,3 inhibitor, valproic acid, was evaluated in a clinical trial in *RAS*-mutated metastatic CRC patients with the aim of improving the treatment efficacy of standard treatment with the VEGF inhibitor bevacizumab and an oxaliplatin/fluoropyrimidine-based chemotherapy regimen (NCT04310176) [189]. However, these studies showed modest therapeutic success.

Considering that HDAC6 is involved in multiple cancer-related pathways and that there was no severe toxicity with HDAC6 inhibition in animal models [154], some therapeutic advantages have been postulated for selective therapeutic targeting of HDAC6. In recent years, intensive investigations have been conducted with the aim of developing small molecular modulators that selectively target HDAC6 and are effective in solid tumors as well as in hematological malignancies. In this sense, several drug candidates have been evaluated in the clinic. HDAC6is tubacin and tubastatin A were the first HDAC6is used in experimental settings [155]. Unfortunately, due to nondrug-like properties such as high lipophilicity, unfavorable pharmacokinetics, and rapid metabolic inactivation, these agents are only used in research as tools to validate HDAC6 as a target but not in clinical settings [155,156,174].

Ricolinostat (ACY-1215) is the first effective selective HDAC6i that became orally available, and it was shown to be ten times more selective against HDAC6 than other HDACis [190,191]. Since its first application in MM [190], ACY-1215 has shown some efficacy in various tumors [192]. By increasing the level of acetylated tubulin, cortactin, and Hsp90, ACY-1215 inhibits cell cycle progression, motility and invasiveness, processing of misfolded proteins, the ubiquitin–proteasome pathway, and autophagy. Furthermore, it renders GRP78 in an acetylated form, thus preventing it from transporting misfolded proteins from the ER lumen, hence increasing ER stress. This effect, together with the increased accumulation of protein aggregates and polyubiquitinated and misfolded proteins on tumor cells, induced apoptosis [193]. Furthermore, ACY-1215 exerts its antitumor effect by the inhibition of proliferation and induction of apoptosis by inhibiting the MAPK/ERK and PI3K/AKT signaling pathways in CRC cell lines [19,168].

Combinations of selective HDAC6is with different alkylating agents have shown synergistic antitumor effects in various tumors including CRC. ACY-1215 showed an antiproliferative effect on CRC cell lines in combination with 5-FU [170]. Also, in combination with oxaliplatin, ACY-1215 promotes cell apoptosis via activation of caspase-3, elevation of the Bak-to-Bcl-xL ratio, and downregulation of p-ERK and p-AKT in CRC cell lines [168]. Collectively, these findings suggest that targeting HDAC6 activity using ACY-1215 may present a promising therapeutic opportunity.

Ryu investigated the anticancer mechanisms of a novel potent and selective HDAC6i, A452, and compared its effect on CRC cell lines with the clinically tested HDAC6i ACY-1215. In the aforementioned study, A452 showed an antiproliferative effect irrespective of p53 status and induced apoptosis by activating caspase-3 and the enzyme involved in DNA repair, poly ADP-ribose polymerase (PARP), increasing Bak and Bax while decreasing Bcl-xL levels. It also inhibited the AKT and ERK pathways and triggered DNA damage by increasing the activation of the checkpoint kinase Chk2, which induces cell cycle arrest and apoptosis [171]. Further in vitro studies indicated that A452 combined with vorinostat was more effective in the induction of apoptosis than either drug alone [172]. Furthermore, some natural products, such as Aceroside VIII, which is a diarylheptanoid isolated from plant Betula platyphylla, were reported to enhance the anticancer activity of the HDAC inhibitor A452 in CRC cell lines [173].

Another selective HDAC6i, citarinostat (ACY-241), is under clinical development for the treatment of relapsed and refractory MM [194] and more recently solid tumors. ACY-241 showed improved antitumor activity in combination with paclitaxel in a mouse xenograft model of ovarian and pancreatic carcinoma [195] that was later confirmed in a phase Ib clinical trial (NCT02551185) conducted in patients with previously treated advanced solid tumors [185].

Nexturastat A (Nex A) is a selective inhibitor of HDAC6 that was first investigated in melanoma and showed antitumor activity in in vitro and in vivo experimental models [151]. This next-generation HDAC6i is unique compared to other selective HDAC6is as it mainly affects immune-related traits and functions in tumors and immune cells with minimal cytotoxic effects [141]. Furthermore, Nex A exerted its antitumor effect mostly by inducing changes in the TME, including decreased infiltration of immunosuppressive cells and upregulation of adhesion molecules such as E-cadherin, thus inhibiting the EMT process as shown in a breast cancer model [196].

MPT0G612 is a novel HDAC6i that has been investigated in experimental in vitro settings and exhibited promising antitumor activity against several solid tumors, including CRC. The antiproliferative and proapoptotic effects of this compound were accompanied by the activation of autophagy mediated with LC3B-II formation and p62 degradation [176].

### 3.1. HDAC6 Inhibition in Combination with Other Therapeutic Modalities

In order to explore the options for the most optimal antitumor activity while minimizing the side effects of the maximally tolerated doses, the selective HDAC6is have been further evaluated in combination with different anticancer agents such as proteasome inhibitors, tyrosine kinase inhibitors, radiotherapy, and immunotherapy.

ACY-1215 has been reported to act more effectively in combination with the protease inhibitor bortezomib, which is currently used in the treatment of MM and Hodgkin’s and non-Hodgkin’s lymphoma [190]. In CRC, ACY-1215 showed an improved antitumor effect in combination with carfilzomib (proteasome inhibitor) by inducing the accumulation of protein aggregates, ER stress, and subsequently apoptosis of treated CRC cell lines harboring the *BRAF*V600E mutation in in vitro and xenograft murine models [169]. Another selective HDAC6i, C1A, also showed potential antitumor activity against *KRAS*-mutated CRC in murine xenograft models [174] in combination with bortezomib that was characterized by inhibited degradation of misfolded proteins in proteasomes and decreased autophagy [175].

*BRAF*V600E mutations that lead to constitutive activation of BRAF kinase and increased RAS/RAF/MEK/ERK signaling have been reported in 10% of patients with CRC [197]. However, in CRC harboring these mutations, response rates to the BRAF inhibitors vemurafenib or dabrafenib and MEK1/2 inhibition with trametinib were low due to adaptive feedback reactivation of upstream RTKs and RAS. In these tumors, BRAF inhibition enhances RAF dimerization and thus results in adaptive feedback reactivation of MAPK signaling, often mediated by EGFR activation [198]. HDAC inhibitors have been tested as potential agents to reduce the resistance to BRAF/MEK inhibition in CRC. Accordingly, one study in a mouse xenograft CRC model and cell lines showed the ability of vorinostat to overcome resistance to treatment with the MEK1/2 inhibitor trametinib. The study also identified a novel resistance mechanism that is mediated via STAT3 and the anti-apoptotic protein c-FLIP [167]. Increasing experimental evidence shows enhanced antitumor effects of the simultaneous application of HDACis and RAF/MEK/ERK-targeting therapeutic agents in other tumors with *BRAF* mutations. The synergistic antitumor effect of vorinostat in combination with BRAF inhibitors [199] and the ability of vorinostat to eliminate BRAF inhibitor-resistant and senescent cells was reported in *BRAF*-mutated melanoma cells [200]. Similarly, panobinostat in combination with MAPK and the BRAF inhibitor dabrafenib has shown synergistic antitumor effects in *BRAF*-mutated thyroid carcinoma cells [201].

As HDAC inhibition often induces cell cycle arrest and inhibits DNA repair, the ability of HDAC inhibitors to improve the therapeutic outcome of radiotherapy and to sensitize the tumor cells to ionizing radiation has been investigated in experimental settings [202]. In this sense, the pan-HDAC inhibitors panobinostat and vorinostat have shown enhanced antitumor effects when used in combination with radiation therapy in clinical trials in prostate and gastrointestinal tract carcinoma [203]. Furthermore, one study reported that treatment with the selective HDAC6i SP-2-225 resulted in decreased tumor growth and increased infiltration of M1 macrophages within tumors. These findings support further investigation of the use of selective HDAC6is to improve antitumor immune responses and prevent post-radiation therapy tumor relapse [204].

### 3.2. Modulation of Antitumor Immunity with HDAC6 Inhibition

HDAC inhibitors have shown considerable immunomodulatory effects by influencing many aspects of the immune response in tumors. However, some differences were reported depending on whether pan-HDACis or selective HDAC6is were used and on the experimental model used in a study [137] (Table 5).

Pan-HDAC inhibitors have been reported to decrease the expression of costimulatory molecules (CD40, CD80, CD83, and CD86) on DCs and secretion of Th1-polarizing cytokines (IL-6, IL-12, TNF) after stimulation of TLRs on DCs that led to the inhibition of T cell activity. Panobinostat reduced the expression of costimulatory molecules on DCs and impaired IFN-γ production by T cells [210]. Similarly, the pan-HDACi vorinostat was reported to inhibit T-cell functions by inducing the transcription of the enzyme indoleamine 2,3-dioxygenase (IDO) in DCs which regulates the catabolism of tryptophan, which is essential for T-cell activation [205]. HDAC inhibition by vorinostat was shown to promote the transcription of IDO through acetylation and activation of STAT3 and hence to inhibit T-cell functions [206]. Contrary to pan-HDACis, selective HDAC6is have shown immunostimulatory effects on DCs. It has been reported that ACY-241 increased the expression of costimulatory and MHC molecules on DCs [222]. Furthermore, it was shown that tubastatin A impairs the production of the immunosuppressive cytokine IL-10 by DCs and macrophages by disrupting the complex between HDAC6 and STAT3 and by impairing STAT3 signaling that in turn increases the production of IFN-γ by CD4 T cells [46,196]. All these factors indicate that HDACis, by affecting APCs, may regulate innate and adaptive immune responses and inflammation in the TME. Moreover, tubastatin A showed an anti-inflammatory effect by inhibiting IL-6 synthesis, nitric oxide (NO) secretion, and cell viability and motility in human macrophages stimulated with LPS [223]. This effect is also relevant for the antitumor effect of tubastatin A, as IL-6 represents a potent EMT-triggering factor in the TME involved in tumor progression, metastatic invasion, and chemoresistance [121].

The effects of pan-HDACis on T-cell activation differ from the effects of specific HDAC6is. The pan-HDACis trichostatin A and rodempsin inhibit the activation-induced proliferation of naïve T cells and IL-2 production [214], unfavorably affect the metabolic reprogramming of recently activated T cells, impair T-cell receptor signaling, and induce T-cell apoptosis [137,215,225]. However, reports on the impact of pan-HDACis on previously activated effector T cells show that when applied after the initial activation of CD4 T cells, the pan-HDACi trichostatin A prevents FasL-driven activation-induced cell death, increases infiltration of CD4 T cells into the tumor, and reduces tumor growth in lymphoma and melanoma murine models [137,217]. Regarding selective HDAC6is, Laino et al. found that peripheral blood T cells of melanoma patients treated in vitro with the selective HDAC6is ACY-1215 and ACY-241 showed decreased production of immunosuppressive Th2 cytokines (IL-4, IL-5, IL-6, IL-10, and IL-13) with concomitant downregulation of the Th2 transcription factor GATA3, upregulated the Th1 transcription factor T-BET, and favored the accumulation of central memory-phenotype T cells. This report indicated the immunostimulating potential of selective HDAC6is on T cells [220].

CD8 T cells directly kill tumor cells by secreting perforin and granzymes and facilitating antitumor immune responses by the production of IFN-γ and TNF, which activate local APCs and increase the immunogenicity of tumor cells by inducing MHC expression and activation of immunoproteasomes. It was reported that treatment with the pan-HDACi vorinostat increased the proliferation and function of CD8 T cells, particularly the frequency of IFN-γ- or perforin-producing T cells in mammary tumor-bearing mice [207]. Contrary to the pan-HDAC inhibitor vorinostat, selective inhibition of HDAC6 may impair the cytotoxic capacity of CD8 T cells, as tumor-specific CD8 T cells from mice treated with the HDAC6-specific inhibitor tubastatin A and the HDAC6-deficient mice showed reduced lytic capacity of CD8 T cells, probably due to interrupted intracellular trafficking and exocytosis of perforin [144]. However, one study reported that another HDAC6-selective inhibitor, ACY-241, increased perforin and IFN-γ production in CD8 T cells [222]. Evidently, due to the conflicting evidence on the effect of different selective HDAC6is on T-cell activity, further investigations are needed [226].

Treatment with selective HDAC6is has been shown to affect the susceptibility of tumor cells to T and NK cell-mediated killing, as they affect the expression of MHC class I molecules on tumor cells. In this sense, the HADC6 inhibitor tubastatin A has been reported to induce the expression of MHC class I molecules on melanoma cells and increase their susceptibility to CD8 T cell-mediated lysis [151]. Moreover, the treatment of colon cancer cells with the pan-HDACi vorinostat increased the expression of death receptor Fas, which led to enhanced Fas-dependent cytotoxic activity of T cells [166]. Furthermore, it has been reported that treatment with the pan-HDACi panobinostat increased the expression of genes involved in cell adhesion and junctions and the formation of conjugates between NK and tumor cells, and modulated the expression of NK cell-activating receptors and ligands on tumor cells, thus contributing to the increased cytolysis of tumor cells [211]. Regarding NK cell antitumor activity, it was reported that the pan-HDACis vorinostat and trichostatin A induced the expression of MHC class I-related chain A (MICA) and B ligands to activate the NK cell receptor NKG2D in hepatocellular carcinoma and Ewing sarcoma and thereby increased the susceptibility of treated tumor cells to NK cell-mediated lysis [209,219]. Furthermore, it was reported that the HDAC6i Nex A increased tumor infiltration with NK cells, an effect that has been associated with improved prognosis and survival in tumors [141,226].

Moreover, some HDACis enhance T-cell migration to the tumor site. The pan-HDACi rodempsin was reported to increase the expression of chemokines CCL5 and CXCL9 and 10 by tumor and stromal cells, increasing tumor infiltration with T cells and thereby improving the antitumor immune response [216].

Selective HDAC6 inhibition contributes to antitumor immunity by inhibiting the differentiation and influx of suppressive immune cells into the TME. Ricolinostat was reported to inhibit the activity of myeloid-derived suppressor cells (MDSCs) and inhibit tumor growth [221], while Nex A treatment has been shown to induce the differentiation of immunostimulating M1 macrophages [141] similar to the pan-HDACi trichostatin A [218].

In summary, the HDAC6is have shown potential inherent to immunomodulatory agents as they have the ability to improve antitumor immunity by stimulating the immunogenicity of tumors and the activity of immune cells.

The expression of immune checkpoint (IC) molecules is often upregulated in immune cells in the TME as a consequence of antitumor immune responses and due to the elevated levels of immunosuppressive mediators produced by tumor cells, suppressive immune cells, and stromal cells [227]. In this sense, in the TME, T and NK cells may express programmed cell death receptor (PD)-1, CTL-4, T-cell immunoglobulin and mucin domain-containing protein 3 (TIM3), lymphocyte activation gene-3 (LAG-3), and TIGIT, which inhibit antitumor immune responses [124,228]. On the other hand, tumor cells often express ligands for ICs that can be induced with oncogenic pathways and extrinsic factors in the TME such as the cytokines IFN-γ, IL-6, and TNF-α [229,230] that contribute to the upregulation of PD-1 ligands 1 and L2 (PD-L1 and PD-L2) on tumor cells. In CRC, the MAPK, PI3K, JAK/STAT3, and phospholipase Cγ signaling pathways have been related to the upregulation of PD-L1 expression [231].

It has been well established that metastases of CRC show an increased expression of PD-L1 compared to primary tumors [232], which is associated with unfavorable disease prognosis [233]. Moreover, the expression of PD-L2 in tumor cells, which is more inherent to immune cells, was recently associated with poor patient survival in CRC [233,234]. Therapeutic blockade of PD-1 and its ligand with anti-PD-1 and anti-PD-L1 antibodies has shown a considerable clinical benefit in some metastatic CRCs [232,235]. However, blockade of PD-1 enhances T-cell function and the subsequent production of inflammatory cytokines, most notably IFN-γ, which enhances PD-L1 and PD-L2 expression on tumor cells, thereby inducing negative feedback as well as other immunosuppressive pathways [227,229,236].

As tumors develop mechanisms allowing them to evade immune responses during their evolution, it is of importance to identify treatments that can increase immunogenicity, minimize immune-related adverse events, and maximize the therapeutic benefits of IC inhibition [124]. Among emerging new therapeutic targets, HDACs have raised great interest, especially HDAC6 since it is involved in the control of immunomodulatory pathways and expression of IC molecules. It has been shown recently that HDAC6 may induce the expression of PD-L1 in cancer cells via the activation and recruitment of STAT3 transcription factors, as shown in experimental models of pharmacological impairment of HDAC6 or by its genetic abrogation in melanoma [224] and breast cancer [196,237]. However, there are differences in existing published data between the effects of some selective pharmacological HDAC6 inhibitors on PD-L1 expression. It was reported that in vitro treatment with Nex A decreased the expression of PD-L1 in melanoma [141] and breast [196] animal tumor models. A similar effect was reported for the in vitro treatment with the novel selective HDAC6i MPTOG612 in CRC cells [176]. However, some publications report the opposing effect for certain selective HDAC6is. One study reported that the treatment with the novel small molecule HDAC6i A452 and ACY-1215 increased PD-L1 expression in CRC tumor cells [171]. Another study reported that ACY-1215 alone and in combination with alkylating agents upregulated PD-L1 in CRC cells in vitro and that was achieved irrespective of p-STAT3 status [168]. However, ACY-1215- and A452-induced PD-L1 expression may increase the susceptibility of tumor cells to PD-1/PD-L1 axis IC blockade therapy.

The use of nonspecific HDACis, such as panobinostat [212] as well as low doses of trichostatin A [218], was reported to increase the expression of PD-L1 and PD-L2 on the cell surface of tumor cells in murine models of melanoma and breast cancer. Moreover, panobinostat was shown to synergize with PD-L1 blockades by different mechanisms such as promotion of NK cell–target cell conjugation formation by increasing the expression of cell adhesion- and tight junction-related genes and by increasing the expression of CD80, CD86 (ligands for CD28), and CD112 (PVRL2/nectin-2 ligand for activating the DNAM-1 NK cell receptor) on tumor cells [211]. These results also indicate the antitumor potential of HDAC inhibition in the context of NK cell-based immunotherapy. It was also reported that panobinostat augmented the expression of MHC I and costimulatory molecules (CD40, CD80) on melanoma cells in vitro, leading to increased activation of antigen-specific T cells [213].

Aside from immune cells, PD-1 is also expressed on malignant cells including colon cancer cells [238]. Moreover, tumor cells expressing PD-1 exhibit a higher ability for proliferation and tumorigenicity [239]. Recently, it has been reported that the transcription of the PD-1 gene in cancer cells is regulated via acetylation of the p53 tumor suppressor by HATs p300, CBP, and Tip60 in a manner that acetylated p53 recruits the acetyltransferase cofactors to interact with the PD-1 promotor and induce the expression of PD-1 [240]. Although validation on a larger sample size is needed, the expression of PD-1 on tumor cells versus its expression on immune cells may have some relevance for IC blockade selection in patients with CRC [238]. However, there are scarce studies on the effect of HDACis on PD-1 in tumor cells [238].

Selective HDAC6 inhibition also affects IC expression on immune cells. In this sense, one study reported that ACY-1215 and ACY-241 decreased the expression of LAG-3, TIM-3, and PD-1 on the peripheral blood T cells of melanoma patients. These findings indicate that inhibition of HDAC6 activity may be effective in alleviating T-cell suppression and enhancement of the cytotoxic function of T cells and may provide a theoretical basis for further evaluation of the potential clinical efficacy of joint HDAC6 and IC inhibition [220]. These effects of HDAC6 inhibition may be relevant for CRC cells as TIM-3 [241], along with PDL-1/2 [207,233], has been identified on tumor cells and has been designated as a negative prognostic biomarker in colon cancer.

### 3.3. Combination Therapies with HDAC6 and IC Inhibitors

Although the IC blockade therapies targeting PD-1 and CTLA-4 have shown considerable therapeutic benefit, they have proved ineffective for some patients, presumably due to the development of resistance to therapy, the immunosuppressive nature of the TME, and lack of antitumor T-cell response prior to therapy. As selective HDAC6 inhibition has shown, in preclinical settings, the ability to alter the expression of PD-L1 and PD-L2 on tumor cells, increase the immunogenicity of tumor cells, induce more effective antigen presentation, relieve T-cell suppression by downregulating the expression of ICs (PD-1, TIM-3, LAG-3) on immune cells [141,217], and reverse the TME by increasing the infiltration of immunostimulating immune cells and inhibiting the differentiation of suppressive immune cells, the combined application of HDAC6i and immunotherapy has recently emerged as a significant approach in the field of cancer treatment [242]. Therefore, the use of HDAC inhibitors to augment antitumor T-cell responses following the application of IC inhibitors may increase the number of patients who respond to IC blockade therapy.

Experimental data obtained on HDAC6 inhibition in combination with immunotherapeutic agents in the settings of tumor cell lines and animal models have shown an improved antitumor effect of this drug combination compared to each agent alone. The examples that support this are investigations of ACY-1215 in combination with anti-PD-L1 therapy in ovarian carcinoma [243], ACY-241 in combination with anti-PD-L1 antibodies in MM [208], Nex A in combination with anti-PD-1 antibodies in melanoma [141], etc. Based on similar and growing experimental data, a considerable number of clinical studies have been conducted in advanced solid tumors. The synergistic effect of the pan-HDACi vorinostat with the anti-PD-1 agent pembrolizumab was shown in metastatic non-small cell lung cancer (NSCLC) patients with good tolerance of both agents in investigated patients [244]. Another phase 2 clinical trial showed that the combined application of vorinostat with pembrolizumab and the ER antagonist tamoxifen in ER-positive breast cancer patients resulted in prolonged progression-free survival of treated patients [245]. A phase 2 trial (Table 4) involving the combined application of pembrolizumab and vorinostat in recurrent metastatic head and neck cancer showed significant efficacy although with a relatively higher grade of toxicity compared with anti-PD-1 monotherapy [246]. Regarding studies on selective HDAC6is in solid tumors, some therapeutic benefit was shown in patients with previously treated advanced NSCLC treated with ACY-241 in combination with the PD-1-blocking drug nivolumab [247].

Regarding CRC, in patients with microsatellite-stable (MSS) CRC who do not respond to IC inhibitors, anti-tumor activity of the HDACi CXD101 and nivolumab (anti-PD-L1) was assessed in a phase II clinical trial (Table 4) and showed good tolerance and efficacy in the treatment of advanced MSS CRC [186]. Furthermore, there is an ongoing phase I clinical study on the joint application of the pan-HDACi romidepsin in combination with the anti-PD-1 agent pembrolizumab in mismatch repair-proficient CRC (Table 4) [184].

Therefore, there is a need for broadening the research on the role of HDACis and IC inhibitors in antitumor treatment to better evaluate their therapeutic potential and possibilities for synergistic application in a wider range of tumors.

### 3.4. Novel HDAC6-Based Therapeutical Approaches

The use of drug combinations can provide efficacy by targeting different signaling pathways and may reverse drug resistance. Although preclinical studies showed that HDACis in combination with other anticancer agents have a better antitumor effect, results obtained in clinical trials have not been in accordance with this. Simultaneous disruption of different signaling pathways and processes aims to reduce tumor growth and induce tumor cell death but unfortunately, some interactions between concomitantly applied drugs cannot be predicted. Desirable additive or synergistic effects of drug combinations may thus impose a risk of inducing adverse effects due to unwanted drug-to-drug interactions [248,249].

Given the advantages and disadvantages of combinational therapies, a novel approach in pharmacology is the design of a drug that can interact with two different targets that are affected by certain diseases. Therefore, new treatments have been developed by conjugating two distinct therapeutic compounds in a single molecule for dual-targeting strategies. This may provide increased efficacy of the drug by targeting additional disease-related pathways. In this sense, dual HDAC and kinase inhibitors have been tested in preclinical and clinical settings [249,250]. At the preclinical level, compounds that interact simultaneously with HDACs and receptor tyrosine kinases including PI3K, Src, CDKs, and JAKs have been developed [248,250,251]. For example, the inhibition of HDACs and PI3K by dual inhibitors in multiple cancer cell lines, including CRC, by regulating both histone and nonhistone substrates can affect a variety of cell functions and synergize with PI3K inhibition [177] (Table 3).

In CRC harboring *BRAF*V600E mutations, which shows a low response rate to BRAF inhibitors due to the emergence of resistance, the novel series of hydroxamate acid- and 2-aminopyridinyl-containing compounds such as BRAF and HDAC dual-targeted inhibitors were investigated. The compounds exerted enzymatic inhibitory activities against BRAFV600E and HDAC1/6 and suppressed the proliferation of CRC cells harboring both wild-type *BRAF* and mutated *BRAF*V600E [178] (Table 3). Therefore, in theory, the optimal pathway blockade can be achieved by simultaneously targeting multiple steps of the pathway.

Other combinations in development include dual inhibitors that contain DNA targeting agents such as the DNA-alkylating agent temozolomide [248]. Furthermore, regarding a dual HDAC6 inhibitor that contains an Hsp90 inhibitor, a compound identified as compound 12 (dual HDAC6 and Hsp90) displayed inhibitory effects toward the HDAC6 isoform and a 246-fold higher selectivity for HDAC6 over HDAC1, 3, and 8 isoforms and was endowed with significant cytotoxic effects against CRC cell lines [173] (Table 3).

As stated before, the multi-target drug design approach aims to enhance drug activity and selectivity and overcome drug resistance. Recently, the proteolysis-targeting chimera (PROTAC) has become a revolutionary technology in modern drug discovery. PROTACs are bifunctional small molecules consisting of an E3 ubiquitin ligase recognition motif and a ligand for the target protein of interest connected by a suitable linker. PROTACs regulate the expression of the target protein of interest at the post-translational level by inducing its degradation in proteasomes [248]. The advantages of PROTAC activity are enhanced selectivity and improved potency. Moreover, this mode of action results in acute post-translational depletion of the pathological protein (target) and eliminates the risk of therapeutic resistance due to physiological feedback mechanisms that upregulate the target protein. In the field of HDAC-targeting PROTACs, HDAC6 was designated as the promising target. Several examples in the literature have demonstrated that HDAC6 could be selectively degraded by converting either HDAC6-specific or even pan-HDAC inhibitors into PROTACs. The first HDAC6 degrader was generated based on a nonselective HDAC inhibitor and pomalidomide as a ligand for the E3 ubiquitin ligase cereblon (CRBN). CRBN ligands rely on the structure of the anticancer drug thalidomide and its derivatives and have proven to be efficient and selective degraders of HDAC6 [252]. This may be due to the cellular localization of HDAC6 or the formation of a more efficient ternary complex [248,252,253]. Until recently, most research on HDAC PROTACs was focused on hematological malignancies that are more sensitive to HDAC6 degraders regarding degradation of HDAC6 [253] although some encouraging results were observed in tumor cell lines derived from solid tumors [254]. However, since this research area has very recently emerged, it is too early to draw any conclusions.

## 4. Concluding Remarks

HDAC6 plays an essential role in many cellular signaling pathways that enable cancer cells to survive and maintain their malignant phenotype. The overexpression of HDAC6 in CRC, its role in promoting tumor growth through regulation of the MAPK/ERK signaling pathway, and its effect on patient survival indicate the oncogenic potential of HDAC6 in this malignancy. In patients with metastatic disease in clinical stage IV of CRC, the currently used therapeutic options have shown limited success due to the emergence of drug resistance and subsequent disease progression. In this sense, considering the role of HDAC6 in metastatic invasion and experimental data showing the potential of HDAC6 inhibition to overcome resistance to targeted therapy, HDAC6 may represent a relevant therapeutic target in metastatic CRC. Furthermore, as selective HDAC6 inhibition increases the immunogenicity of tumor cells, effective antigen presentation, and immune cell functions and reduces immunosuppression in the TME, HDAC6 inhibitors could potentially improve the antitumor immune response in the context of therapy with IC inhibitors in metastatic CRC tumors. Further studies should be focused on the improvement of HDAC6 inhibitors to increase their selectivity and accumulation in solid tumors so that adverse effects exerted by high doses on healthy cells would be minimized. Novel approaches in drug design, such as dual inhibitors targeting BRAF and HDAC6, may represent good candidates for further evaluation in the therapy of metastatic CRC with *BRAF*V600E mutations. Further investigations and development of novel HDAC6-based bifunctional inhibitors and PROTACs that target HDAC6 in combination with other molecular targets involved in angiogenesis, cell cycle regulation, and oncogenic signaling, may lead to their therapeutic evaluation in metastatic CRC in the future.

## Figures and Tables

**Table 1:** HDAC6 substrates and physiological functions of HDAC6-mediated deacetylation.

Protein	Localization	Function	Reference
α-tubulin	Cytoplasm	Microtubule disassembly, increases cell motility	[26,27,28,29]
Cortactin	Cytoplasm	Actin polymerization and branching, increases cell motility	[30]
TFEB	Cytoplasm	Autophagy	[31]
FOXO1	Cytoplasm	Autophagy	[32]
Hsp90	Cytoplasm	Degradation of misfolded proteins	[33]
GRP78	Cytoplasm and nucleus	ER stress regulation, tumor progression via secretion of exosomes	[34]
NF-κB	Nucleus	Transcription of genes for NLRP3, pro-IL-1β, pro-IL-18, inflammasome activity	[35]
P53	Cytoplasm	Cell cycle progression, inhibition of apoptosis, induced autophagy via upregulation of Beclin-1	[36,37,38]
Ku70	Cytoplasm	Suppression of apoptosis	[25,39]
Survivin	Nucleus	Suppression of apoptosis	[40,41]
Peroxidins I and II	Cytoplasm and nucleus	Antioxidant activity	[42]
Smad3	Cytoplasm	Downregulation of E-cadherin expression, EMT	[43,44]
β-catenin	Cytoplasm	Translocation into nucleus and tumor cell invasion	[45]
STAT3	Cytoplasm	Activation of JAK/STAT3 signaling and inflammatory responses	[46]
TAK1	Cytoplasm	Activation of ADAM17 MMP enhances sIL-6R release and M2 macrophage differentiation	[47]
ERK1	Cytoplasm	Activation of ERK1, proliferation, survival, and increased cell motility	[48]
AKT	Cytoplasm	Activation of AKT pathway, cell migration	[49]

TFEB—transcription factor EB, Hsp—heat shock protein 90, GRP—glucose-regulated protein 78, ER—endoplasmic reticulum, NLRP3—NLR family pyrin domain-containing 3, EMT—epithelial–mesenchymal transition, JAK—Janus kinase, STAT—signal transducer and activator of transcription 3, ADAM—A disintegrin and metalloproteinase-17, MMP—matrix metalloproteinase, sIL-6R—soluble IL-6 receptor, TAK—Transforming Growth Factor-β-activated kinase 1, STAT—signal transducer and activator of transcription 3, ERK—extracellular signal-regulated kinase 1.

**Table 2:** Proteins that interact with HDAC6 by nonenzymatic interactions.

HDAC6 Domain	Protein	Function	References
DMBS	Dynein/p150glued	Aggresome formation and autophagy	[27]
ZnF-UBP	Ubiquitin chain	Protein degradation	[52]
Not defined	TRIM50	E3-Ubiquitin ligase activity promotes recruitment of polyU proteins to aggresome and degradation	[53]
Not defined	P97/VCP	Dissociation of HDAC6 and polyU protein and protein delivery to proteasomes	[54,55]
Not defined	HSF1	Release of HSF1 and transcription of genes for Hsp90 and Hsp70 molecular chaperons	[56,57]
Not defined	Runx2	Proliferation, inhibition of apoptosis	[58]

DMBS—dynein motor-binding sequence, ZnF-UBP—zinc finger ubiquitin-binding domain, U—ubiquitin, PolyU—polyubiquitinated, HSF—heat shock factor 1, Hsp—heat shock protein.

**Table 3:** HDAC6-inhibiting agents and their effects in colorectal cancer evaluated in preclinical studies.

Inhibitor	Effect	Reference
**Pan-HDAC inhibitors**		
Vorinostat (SAHA)	Inhibition of proliferation, downregulation of mutated p53, upregulation of wtp53, inhibition of HDAC6–Hsp90 axis, apoptosis	[39,99,164]
Vorinostat + 5-fluorouracil	Inhibition of proliferation, downregulation of mutated p53	[164]
Vorinostat + decitabine	Inhibition of proliferation and migration, apoptosis, decreased pMEK and pERK	[166]
Vorinostat + trametinib	Inhibition of proliferation, apoptosis	[167]
Vorinostat + trichostatin A	Attenuation of Wnt signaling, apoptosis	[165]
Trichostatin A	Increased acetylation of Ku70 and apoptosis by releasing Bax	[104]
**Selective HDAC6 inhibitors**		
ACY-1215	Inhibition of MAPK/ERK and PI3K/AKT signaling; acetylated tubulin, cortactin, Hsp90, and GRP78	[168]
ACY-1215 + oxaliplatin	Apoptosis, downregulation of p-ERK and p-AKT	[168]
ACY-1215 + carfilzomib	Accumulation of protein aggregates, ER stress, apoptosis	[169]
ACY-1215 + 5-fluorouracil	Inhibition of proliferation	[170]
A452	Activation of caspase-3 and PARP; increased Bak and Bax, decreased Bcl-xL level, increased PD-L1 expression	[171]
A452 + Vorinostat	Inhibition of proliferation, apoptosis	[172]
A452 + Aceroside VIII	Inhibition of proliferation, apoptosis	[173]
C1A	Inhibition of proliferation, apoptosis acetylation of α-tubulin and HSP90	[174]
C1A + bortezomib	Accumulation of misfolded proteins and decreased autophagy	[175]
Tubacin	Ku70 acetylation and suppression of FLIP, apoptosis	[39]
MPT0G612	Inhibition of proliferation, apoptosis, decreased PD-L1 expression	[176]
**Dual HDAC inhibitors**		
CUDC-907	Inhibition of HDAC6 and PI3K signaling	[177]
compound 12	Inhibition of HDAC6 and Hsp90	[173]
compound 14b	Inhibition of HDAC6 and BRAF signaling	[178]

Hsp—heat shock protein 90, pMEK—phosphorylated mitogen-activated protein kinase, pERK—phosphorylated extracellular signal-regulated kinase, MAPK—mitogen-activated protein kinase, ER—endoplasmic reticulum, PARP—poly (ADP-ribose) polymerase, PI3K—phosphoinositide 3-kinases, PD-L1—programmed death-ligand 1.

**Table 4:** Clinical trials evaluating HDAC6 inhibition in colorectal cancer.

Clinical Study	Phase	NCT Number/Reference
Vorinostat + 5-fluorouracil	Phase I/II	[181]
Vorinostat + 5-fluorouracil + leucovorin calcium + oxaliplatin	Phase I	[182]
Vorinostat + 5-fluorouracil + leucovorin calcium	Phase II	NCT00942266 [183]
Romidepsin	Phase II	NSC-630176, [180]
Romidepsin + pembrolizumab	Phase I	NCT02512172, [184]
ACY-241 + paclitaxel	Phase Ib	NCT02551185, [185]
CXD101 + nivolumab	Phase II	[186]

**Table 5:** Immune-related effects of HDAC6 inhibition.

	Cell	Effect	References
**Pan-HDAC inhibitor**			
Vorinostat (SAHA)	DCs	↓CD40, CD80, CD83,	[205]
		↓TNF, IL-6, IL-12	[205]
		↑IDO	[205,206]
	T cells	↑T cell proliferation	[149,195]
		↑cytotoxicity, IFNγ	[207]
		↑Fas-mediated cytotoxicity	[166]
	Tumor cells	↑PD-L1	[208]
		↑MICA/MICB (NK cell ligands)	[209]
Panobinostat	DCs	↓CD40, CD83, ↓MHC I	[210]
(LBH589)		↓TNF, IL-6, IL-10, IL-12, IL-23	[210]
	CD4 T cells	↓IFN-γ	[210]
	Tumor cell	↑CD80, CD86, CD112(↑ NK cell synapsis)	[211]
		↑PD-L1,	[211,212]
		↑MHC I, CD40, CD80	[213]
Rodempsin	T cells	↓proliferation, activation	[214]
		↑apoptosis	[215]
	Tumor cells	↑CCL5, CCXL9,10	[216]
Trichostatin A	Naïve T cells	↓T cell proliferation, activation	[214,217]
		↑T cell infiltration, ↑ apoptosis	[217]
	Macrophages	↑M1 differentiation	[218]
	Tumor cells	↑MICA/MICB	[219]
		↑PD-L1	[218]
**HDAC6 inhibitor**			
Ricolinostat (ACY-1215)	T cells	activation (CD38)	[220]
		↑perforin, IFN-γ/IL-2	[220]
		↓PD-1, TIM3, LAG-1	[220]
		↓IL-4, IL-5, IL-6, IL-10, IL-13	[220]
	MDSC	↓MDSC	[221]
	Tumor cells	↑CD80, CD86, MHC I, MHC II	[168]
		↑PD-L1	[168,171]
Citarinostat (ACY-241)	Tumor cells and DCs T cells	↑CD80, CD86, MHC I, MHC II	[222]
		↑costimulatory (CD28, 41BB, CD40L, OX40)	[222]
		↓IL-4, IL-5, IL-6, IL-10, IL-13	[220]
		↑perforin, IFN-γ/IL-2	[220,222]
		↓PD-1, TIM3, LAG-1	[220]
Tubastatin A	DCs	↓IL-10	[46]
	T cells	↓perforin secretion	[144]
	Macrophages	↓TNF, IL-6, NO	[223]
	Tumor cells	↑MHC I	[151]
	Treg	↑FoxP3, CTL-4, IL-10, PD-1	[146]
Tubacin	Treg	↑FoxP3, CTL-4, IL-10, PD-1	[146]
Nexturastat A	NK cells	↑NK cell infiltration	[141]
	macrophages	↑M1 differentiation	[141]
	Tumor cells	↓PD-L1	[141,196,224]
MPTOG612	Tumor cells	↓PD-L1	[176]
A452	Tumor cells	↑PD-L1	[171]
SP-2-225	Macrophages	↑M1 differentiation	[204]

DCs—dendritic cells, MDSC—myeloid-derived suppressive cell, NK—natural killer cell, TNF—tumor necrosis factor, IL—interleukin, IDO—indoleamine 2,3-dioxygenase, IFN—interferon, MHC—major histocompatibility class molecule, MIC—MHC class I polypeptide–related sequence-A/B, NO—nitric oxide, PD—programmed cell death protein 1, L—ligand, CTL—cytotoxic T lymphocyte-associated protein 4, TIM—T-cell immunoglobulin and mucin domain-containing protein 3, LAG—lymphocyte activation gene-1.

## Data Availability

Not applicable.
